# Japan prefectural emission accounts and socioeconomic data 2007 to 2015

**DOI:** 10.1038/s41597-020-0571-y

**Published:** 2020-07-13

**Authors:** Yin Long, Yoshikuni Yoshida, Haoran Zhang, Heran Zheng, Yuli Shan, Dabo Guan

**Affiliations:** 1 Institute for Future Initiatives, University of Tokyo, 7-3-1 Hongo, Bunkyo-ku, Tokyo 113-8654 Japan; 2 Research Group of China Emission Accounts and Datasets (CEADs), Beijing, China; 3 Graduate School of Engineering, University of Tokyo, 7-3-1 Hongo, Bunkyo-ku, Tokyo 113-8654 Japan; 4 Center for Spatial Information Science, The University of Tokyo, 5-1-5 Kashiwanoha, Kashiwa, Chiba 277-8563 Japan; 5 School of International Development, University of East Anglia, Norwich, NR4 7TJ UK; 6 Integrated Research for Energy, Environment and Society (IREES), Energy and Sustainability Research Institute, University of Groningen, Groningen, 9747 AG Netherlands; 7 Department of Earth System Science, Tsinghua University, Beijing, 100084 China

**Keywords:** Energy policy, Climate-change mitigation, Environmental impact

## Abstract

In the wake of the Fukushima nuclear disaster, Japan largely moved away from nuclear power generation and turned back towards an energy sector dominated by fossil fuels. As a result, the pace towards reaching emission reduction targets has largely slowed down. This situation indicates that higher emissions will continue to be generated if there is no appropriate and efficient measurement implemented to bridge the energy demand gap. To contribute adequate mitigation policies, a detailed inventory of both CO_2_ emissions and socioeconomic factors, both at the national and regional level, should be issued. Thereby, this work contributes to a time-series emission with a record of 47 prefectures in Japan as well as their associated socioeconomic features. The compiled emission inventory is based on three major fossil fuels and 26 sectors with careful emission allocations for regional electricity generation. This dataset is uniformly formatted and can be expected to provide vital information to set regional reduction allowances and sectoral reduction priorities.

## Abstract (summary)

**Table:** 

Measurement(s)	carbon dioxide emission • Socioeconomic Indicator
Technology Type(s)	fossil fuel power plant • digital curation
Factor Type(s)	prefectural carbon emission
Sample Characteristic - Environment	anthropogenic environment
Sample Characteristic - Location	Japan

Machine-accessible metadata file describing the reported data: 10.6084/m9.figshare.12487604

## Background & Summary

Greenhouse-gas emissions (GHGs) are already committing the planet to likely climate changes in the next 20 years^1^, with fossil fuel combustion expected to release the most substantial amount of GHGs. Over the past few decades, the international community has adopted a series of commitments and agreements aimed at achieving sustainable development through cross-boundary collaboration^2^. These include the Kyoto Protocol with quantified targets for reductions in emissions of GHGs set for each Annex I Parties respectively; and for Paris Agreements reached in 2015, aiming to achieve net-zero emission of anthropogenic GHGs by the second half of this century, which has come up with different national reduction goals and is expected to contribute to the international goal as a whole. As one of the most developed countries, Japan has ratified the Paris Agreement and has pledged an absolute reduction in its emissions by 26.0% by fiscal year (FY) 2030 (Compared with FY2013), which is one of the ambitious climate pledges from intended nationally determined contributions (INDCs)^3–6^. However, in the wake of the 2011 Fukushima nuclear disaster, Japan largely moved away from nuclear power generation and turned towards an energy sector dominated by fossil fuels (See Appendix Figs. 1 and 2). The adverse consequences following the disaster increase the difficulty of reaching the reduction target. Within two years of the Fukushima nuclear disaster, Japan’s national emission reached 1410 million tons of CO_2_ equivalent (Mt-CO_2_eq) in FY 2013, which reached 2.0% increase based on the FY2005. In the FY2017, the total GHGs of Japan is estimated as 1,292 Mt-CO_2_eq, with 90% of emissions found to be carbon dioxide (CO_2_)^7^. Undoubtedly, the Fukushima nuclear accident shows the weakness of Japan’s energy mix and has evolved into an obstacle for future social decarbonization. Given this, Japan has been the subject of worldwide focus concerning its resilience from disaster and the secondary GHGs increase.

On the other hand, given the essential role of emission accounting, an emission inventory enables follow-up research to come up with social decarbonization actions from a multi-disciplinary perspective. Quantification of fossil fuel CO_2_ emissions at high space and time resolution is emerging as a critical need in carbon cycle and climate change research^8^. Furthermore, sub-national inventories are vital for various levels of decision-making^9–11^, prioritizing reductions^12,13^ and volume targets^14^. There is an extensive body of literature on emission accounting at regional level^15,16^, or to a lesser extent at prefectural^17^, city-level^18–23^ or higher resolution supported by remote sensing technology^8^. Previous efforts demonstrate that a detailed scale of inventory will enhance our understanding of regionality and spatial heterogeneity, which has been emphasized in previous sub-national accounting studies^9,10,12,24–26^. These studies demonstrate a recent trend toward sub-national emission accounting, which manifest in increasing attention on the societal demand for detailed emission information^9,27–29^.

Therefore, to realize the reduction targets by FY2030 and promote an efficient reduction mechanism in the long-term, a means of accurate emission accounting is the first, as well as an essential, step to achieving the decarbonization target. Given this, previous studies have tried to analyze regional energy consumption and discussion of regional emission responsibility by a single year in Japan. However, the system boundary varied by research target and data availability with inconsistent estimates, which would be likely to lead to misleading conclusions^30–32^. The discrepancy among different fiscal years cannot now support a continuous observation of regional energy and emissions.

In Japan, the Ministry of the Environment (MoE) and the National Institute for Environmental Studies (NIES) have released Japanese National Greenhouse Gas Emissions data by each fiscal year. Similarly, the International Energy Agency (IEA) and Carbon Dioxide Information Analysis Centre (CDIAC) also provide national scale GHG emissions of Japan, but they vary from each other’s estimation. Furthermore, according to our observations in previous estimates, all the existing emission inventories only present Japan’s total CO_2_ emissions, with regional emission details missing. There is scarcely any emission database constructed according to detailed sectors for Japan and its 47 prefectures. Furthermore, there is no comprehensive emission and socioeconomic dataset which keeps unified sectoral classification.

To bridge to this data gap, the dataset firstly estimated by this study presents the CO_2_ emission inventory by three major fossil fuel for 25 sectors (Except Electricity sector), according to regional sectoral energy consumption statistics. In this data, the three emission sources are then classified as non-power use coal, non-power use crude oil and non-power use natural gas. The Electricity sector is estimated separately according to electric company and power plant data. Thus, the total prefectural emission of 26 sectors can be generated. In addition, the socioeconomic dataset is constructed by unified format and constant price of 2011. This dataset can be easily utilized by both national and regional emission structure analysis and the driving force tracking it.

## Methods

### Sectoral emission accounting

The estimated scope is defined by covering four major sectors in Japan, which entails industrial sectors, household sector, government sectors, as well as other non-energy source sectors, totally 26 sectors. The original data are driven from sectoral energy consumption for the Natural Resource and Energy of Japan by prefectural scale. Each sector contains fossil fuel consumption data entailing coal, crude oil and natural gas. To the best of our knowledge, Japan has no fuel-specific data for electricity generation at prefecture level. As there is a lack of the prefectural power plant fuel-specific emission, the electricity-based emission is listed along with three major fuels (non-power use). Therefore, apart from territorial consumption on non-power use coal, non-power use crude oil and non-power use natural gas, we allocate the emission released by power plants without further divided into three fossil fuels. In other words, the fourth emission source is treated as a single emission source by considering total emission generated by fossil fuel for territorial electricity generation. With the exception of the electric generation industry, the other 25 industries can all be estimated by non-power use fuel-specific data.

Consequently, a down-top accounting and allocation method is applied. We collected the regional power plant and extract capacity data from Japan National Land Numerical Information (Category: Facilities), which is prepared for the latter inter-region emission allocation. Please note, that although energy consumption data are recorded fuel-specifically, the electricity regional power plant can only provide the total emission by referring to the regional electric companies. The companies are basically supported the local, and part of the surrounding prefectures. Combined with the three fossil fuels, the sectoral emission by each prefecture can be expressed as:

1 $$E{L}_{kt}=E\_rati{o}_{kt}\times {\sum }_{i=1}{P}_{{\rm{\varnothing }}kt}^{i}\times {J}_{it}\times {C}_{it}$$

2 $${E}_{ijkt}={U}_{ijkt}\times {J}_{it}\times {C}_{it}$$

3 $${E}_{kt}=\sum _{i=1}\sum _{j=1}{E}_{ijkt}+E{L}_{kt}$$

where *EL*_*kt*_ is the emission of electricity generation by prefecture *k* in year *t*. $E\_rati{o}_{kt}$ refers to prefecture *k*’s power plant capacity ratio in it located region which is supported by electric power company ∅. This parameter is driven from the prementioned Japan National Land Numerical Information (Category: Facilities). ${P}_{\varnothing kt}^{i}$ indicates the prefecture *k*’s supporting electric power company ∅’s electricity generation (kilowatt-hour) on power use fuel type *i* in year *t*. The ${P}_{{\rm{\varnothing }}kt}^{i}$ is driven from Electricity Statistics Information of The Federation of Electric Power Companies of Japan (FEPC). *J*_*it*_ represents the thermal value based on one unit of consumption in the year *t* and *C* is corresponding emission intensity. Here, *J*_*i*_ and *C*_*i*_ value is driven from the Prefecture Energy Statistics of Agency for Natural Resources and Energy of Japan. Fuel type based caloric value and emission factor is shownby Table 1. Here, each value is given by year, which is provided by the Agency for Natural Resources and Energy of Japan. Therefore, ${\Sigma }_{i=1}\,{P}_{\varnothing kt}^{i}\times {J}_{it}{C}_{it}^{e}$ can be understood as each region’s total emission from power use coal, power use crude oil and power use natural gas. Noted that Eq.(1) is only for prefectural electricity generation. Although the regional total electricity generation can be estimated by the three fossil fuels, the power plant inside each prefecture lacks fuel-specific information. Therefore, *EL*_*kt*_ indicates the total emission of prefecture *k*’s electricity generation regardless of its fuel details In Eq.(2), *E*_*ijkt*_ indicates the total carbon dioxide emission generated by the combustion of fuel *i* in prefecture *k*’s sector *j* in the year *t*. *U*_*ijkt*_ presents the consumption data on fuel *i* in prefecture *k*’s sector *j* in the year *t*. The *Uijt* is driven from the Prefecture Energy Statistics of Agency for Natural Resources and Energy of Japan. Equation(3) is the prefectural sector-specific emission in prefecture *k* of year *t*.

**Table 1:** Fuel types and corresponding caloric value by year.

Fuel types of this study (*i*)	Fuels in Japan Prefecture Energy Statistics	Unit	Year (*t*)	*J*_*i*_	*C*_*i*_
				$\frac{{\bf{1}}{{\bf{0}}}^{{\bf{12}}}\,{\boldsymbol{J}}{\boldsymbol{o}}{\boldsymbol{u}}{\boldsymbol{l}}{\boldsymbol{e}}}{{\bf{M}}{\bf{e}}{\bf{a}}{\bf{s}}{\bf{u}}{\bf{r}}{\bf{i}}{\bf{n}}{\bf{g}}\,{\bf{U}}{\bf{n}}{\bf{i}}{\bf{t}}}$	$\frac{{\boldsymbol{t}}{\boldsymbol{o}}{\boldsymbol{n}}\,{\boldsymbol{o}}{\boldsymbol{f}}\,{\boldsymbol{c}}{\boldsymbol{a}}{\boldsymbol{r}}{\boldsymbol{b}}{\boldsymbol{o}}{\boldsymbol{n}}}{{\bf{1}}{{\bf{0}}}^{{\bf{12}}}\,{\boldsymbol{J}}{\boldsymbol{o}}{\boldsymbol{u}}{\boldsymbol{l}}{\boldsymbol{e}}}$
Coal		10^3^ t	2007	25.7	24.6
			2008	25.7	24.6
			2009	25.7	24.6
			2010	25.7	24.6
			2011	25.7	24.6
			2012	25.7	24.6
			2013	26.0	24.5
			2014	26.0	24.5
			2015	26.0	24.5
Crude Oil		10^3^ kl	2007	38.1	18.0
			2008	38.2	19.4
			2009	38.1	18.4
			2010	38.2	18.4
			2011	38.2	17.3
			2012	38.1	18.3
			2013	38.2	18.3
			2014	38.0	18.3
			2015	38.2	18.3
Natural Gas	Natural Gas	10^3^ t	2007	54.5	13.9
			2008	54.8	13.9
			2009	54.6	13.9
			2010	54.6	13.9
			2011	54.7	13.9
			2012	54.7	13.9
			2013	55.0	13.8
			2014	54.5	14.0
			2015	54.5	14.0
	Town Gas	10^6^ m^3-^STAP	2007	44.8	13.9
			2008	44.8	14.0
			2009	44.8	13.9
			2010	44.8	14.1
			2011	44.8	14.1
			2012	44.8	14.0
			2013	40.3	14.1
			2014	42.5	14.1
			2015	42.2	14.2

### Sectoral consumption on electricity and its allocation

**Fig. 1:**
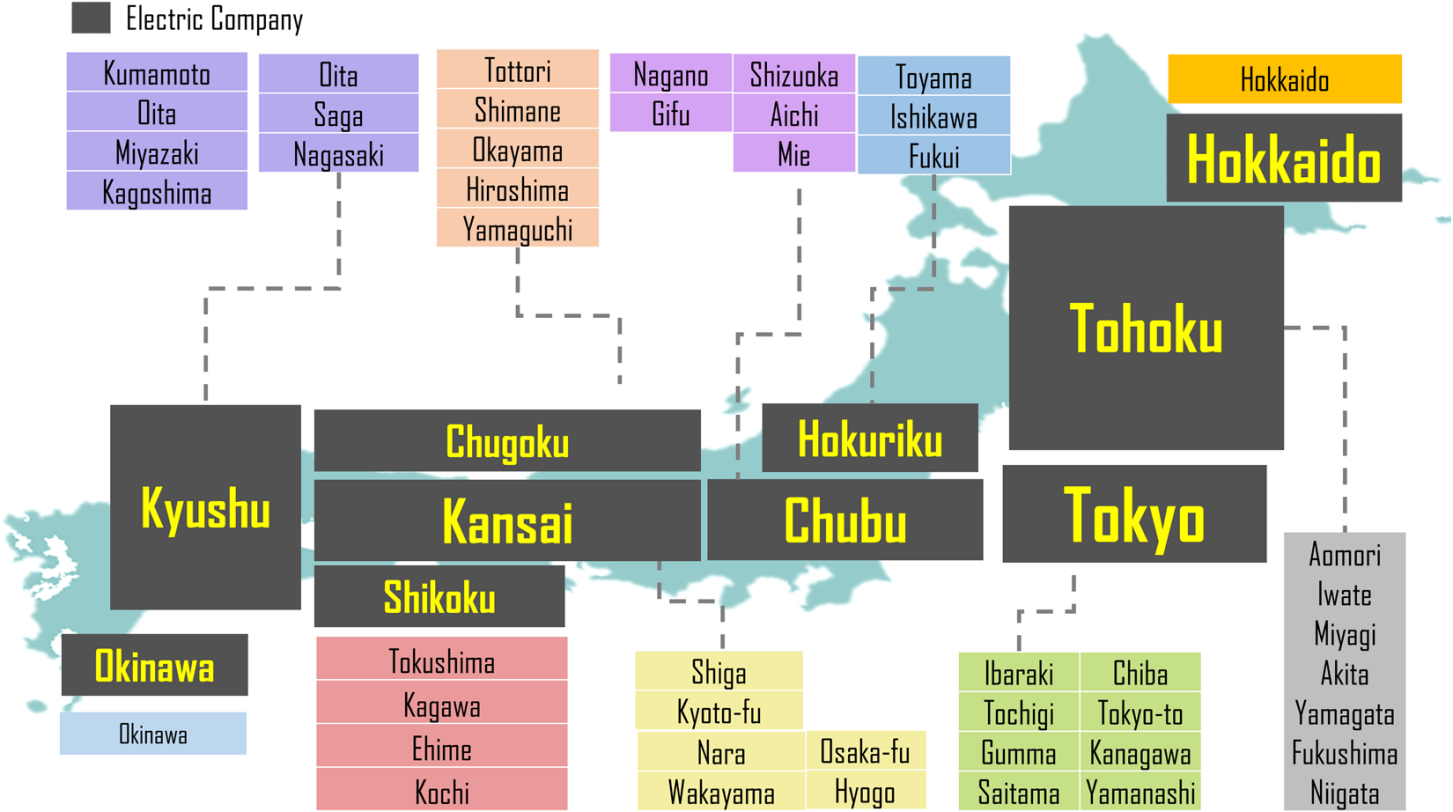
Ten electric companies and their major coverage.

As aforementioned, based on the energy statistics of Japan’s prefecture, energy-related emission accounting is recorded by total final consumption on fossil fuel and electricity. This dataset focuses on sectoral energy consumption and coal, coal products, oil, oil production and natural gas, which can be further condensed to coal, crude oil and natural gas. To cope with this missing data of fuel-specific combustion at prefectural electricity generation, we firstly collected energy consumption for power use from 10 major electric companies based on a selected period. Finally, the emission is reallocated according to a local power plant’s capacity and attributed to prefectural emission accounting for electricity generation. Fig. 1 shows the coverage of the ten power plants across Japan. In some cases, one prefecture is supported by two power companies and in this situation, we recognize the power company giving the larger coverage to be defined as the major supporter.

### Socioeconomic data

Apart from the emission data, we further compile the corresponding socioeconomic indicators to match each item of emission results. The socioeconomic indicators include Population, Gross Domestic Product (GDP) and Per capita Revenue. The constant value of 2011 is adopted. The Population data is referenced from The Statistics Bureau of Japan by each prefecture from 2007 to 2015. Prefectural GDP is derived from the Annual Report on Prefectural Accounts of Cabinet Office, Japan. Socioeconomic indexes are recorded as follows:

1. Population (Unit: People)
2. GDP (Millions of Japanese Yen, constant-price of 2011 is applied)
3. Per capita Revenue (Millions of Japanese Yen, constant-price of 2011 is applied)
4. GDP Deflator (Changes in prices for all of the goods and services produced in an economy at the base year of 2011)

### Data records

**Fig. 2:**
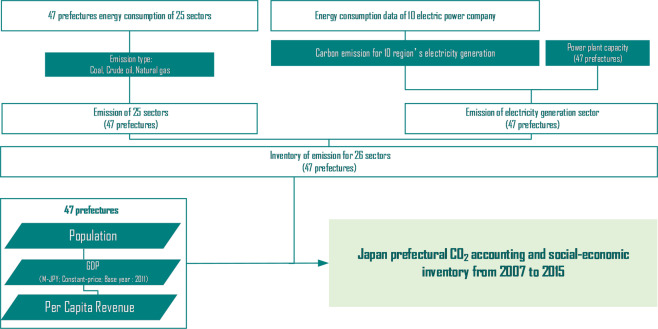
Flowchart of this dataset compiling.

The emission inventory contains 30,456 data records, 47 prefectures, 26 sectoral (See Table 2) energy consumption based on three fossil fuel types, and one secondary energy (Electricity) covering nine years. This dataset is made public under Figshare^33^ which named as “Japan prefectural CO_2_ accounting and social-economic inventory from 2007 to 2015”. Besides, the socioeconomic inventory contains 1,701 data record covering four major indexes. The flowchart of this dataset is shown as Fig. 2.

**Table 2:** Sectoral details.

No.	Sector name
1	Agriculture, Forestry and Fishery
2	Mining, Quarrying of Stone and Gravel
3	Construction Work Industry
4	Manufacture of Food, Beverages, Tobacco and Feed
5	Manufacture of Textile Mill Products
6	Manufacture of Pulp, Paper and Paper Products
7	Printing and Allied Industries
8	Manufacture of Chemical and Allied Products, Oil and Coal Products Manufacture of Plastic Products, Rubber Products and Leather Products
9	Manufacture of Ceramic, Stone and Clay Products
10	Manufacture of Iron and Steel
11	Manufacture of Machinery
12	Manufacture of Lumber, Wood Products, Furniture and Fixtures Miscellaneous Manufacturing Industry
13	Electricity, Gas, Heat Supply and Water
14	Information and Communications
15	Transportation and Postal Activities
16	Wholesale and Retail Trade
17	Finance and Insurance
18	Real Estate and Goods Rental and Leasing
19	Scientific Research, Professional and Technical Services
20	Accommodations, Eating and Drinking Services
21	Education, Learning Support
22	Medical, Health Care and Welfare
23	Living Related and Personal Services and Amusement Services Compound Services Miscellaneous Services
24	Government
25	Residential
26	Non-Energy

## Technical Validation

### Total sectoral emission by energy sources

**Fig. 3:**
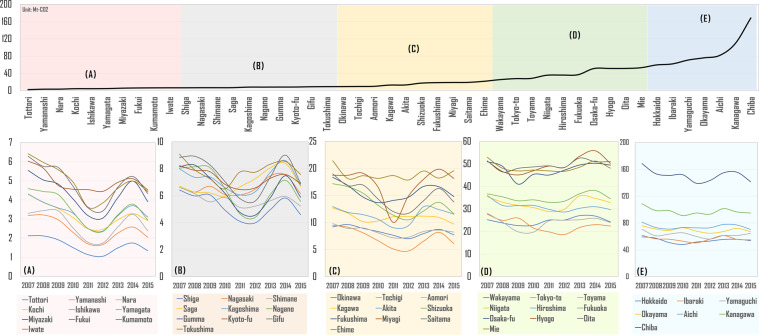
Prefectural total carbon dioxide emission from 2007 to 2015. The upper line chart is sorted by average emission level of 47 from lowest to highest, which can be divided into prefectural groups from A to E. The five lower line charts show the emission fluctuation by group classification and corresponding color.

**Fig. 4:**
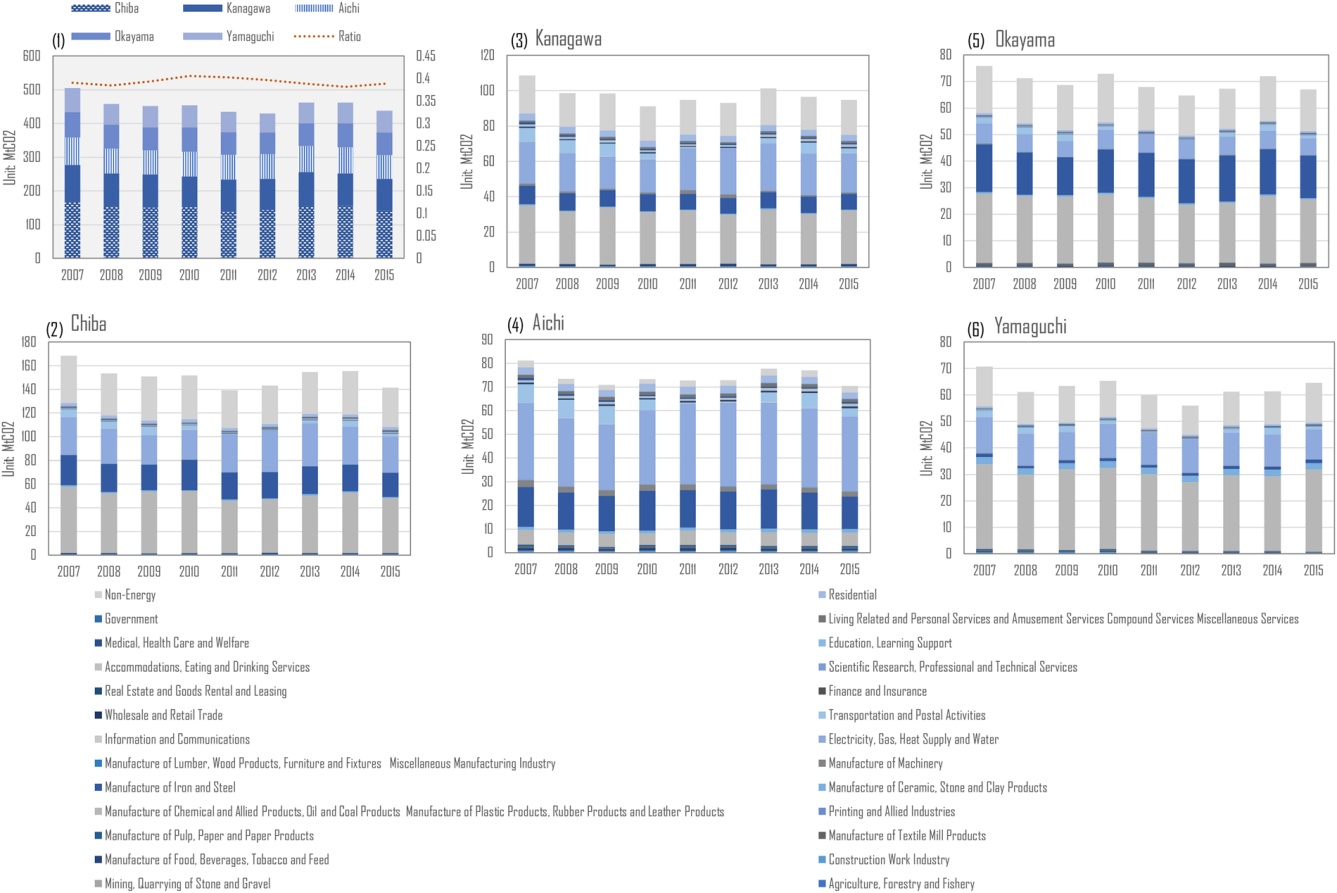
Ratio and emission details of top five regions in Japan.

Figure 3 gives out the annual emission volume from 2007 to 2015, which shows an apparent fluctuation after 2011 with a high level of emission found in Chiba, Kanagawa, Aichi, Okayama and Yamaguchi. The evaluation revealed by this indicates the top five emissions. Further understanding of the role of aforementioned five areas is given in Fig. 4 which gives out the detailed emission information of that. Fig. 4 entails six sub-charts in which Fig. 4-(1) firstly shows the total emission and total share of national emission volume. The result shows that although the total emission drops around 2011, the ratio shows a slightly increasing trend. During the observed years, those top five regions continuously account for 40% of national emissions. Then, Fig. 4-(2) to (6) highlight the emission structure by sectors. For example, emissions from Aichi are found to be mostly contributed by electricity generation. In 2011 and 2012, carbon emission from electricity generation of Aichi was 34.07 and 5.33 MtCO_2_, respectively, which has increased by around 2.5 MtCO_2_ from 2007.

### Comparison with other estimation results

**Fig. 5:**
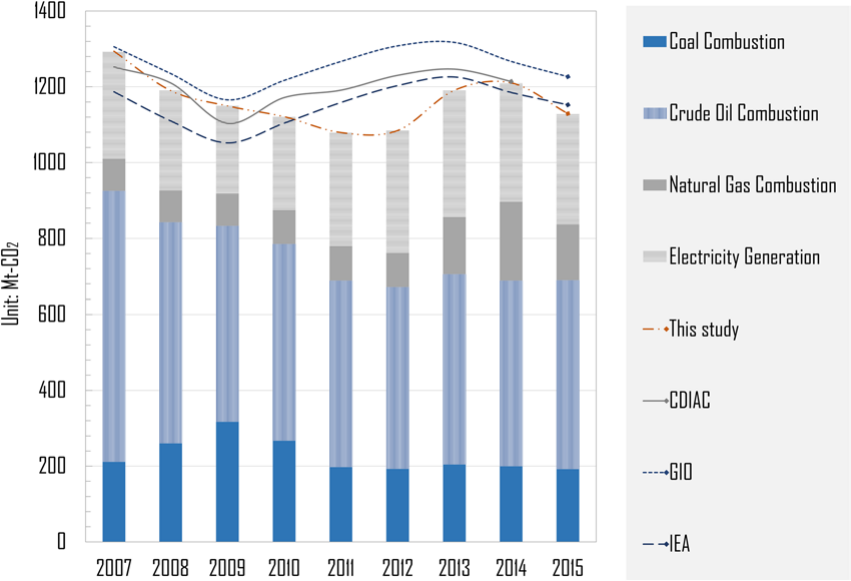
Comparison with other estimation results.

The system boundary of this dataset is based on territorial consumption. The compilation of the industrial energy and emission inventory is based on the direct energy consumption by each sector. Here, we compared the calculated results with the Greenhouse Gas Inventory of Japan (GIO), IEA and CDIAC (see Fig. 5) and found some difference. For example, the GIO difference ratios (Evaluation gap/GIO value) are 0.16% (2007), 1.1% (2008), 6.6% (2009), 5.8% (2010), 3.6% (2011), 8.2% (2012), 6.6% (2013), 4.7% (2014) and 0.16% (2015), respectively. And the largest difference compared with the other national estimates is observed around FY 2011. And the reason causing this evaluation gap is found to be electricity generation. After Great East Japan Earthquake, burden of electricity generation caused by suspension of nuclear power plant has been transferred to energy conservation actions and also partially to private power generation (non-utility generation facility). Those private facilities would only be operated in case of emergence. Therefore, the generation capacity and fuel combusted by private power facilities are difficult to be investigated or quantified accurately. Given this, prefectural generation estimates of this study do not include private power generation, which generated the evaluation gap with other national-level estimation around FY 2011.

### Uncertainties, limitations and future work

In this study, the compilation of prefectural emission inventory is based on sectoral energy consumption. The datasets have several limitations and have led to more uncertainty. The first limitation is the allocation of electricity. Although this study has conducted a survey based on the geographical location of each prefecture, the real electricity supply may be different to some extent, such as in the case of one prefecture being partially supported by two electric companies. The situation of region-crossing electricity supply may exist and further affect the estimated results. Second, the private power generation is not included, and this uncertainty may induce the total emission bias, compared with other national emission estimations. Current days, nearly half of Japan’s electricity generation relies on gas consumption, and natural gas is chosen to be the largest contributor to fill the gap created by the closed nuclear plant. However, the Japanese government expects the natural gas supply to drop to 27% by the fiscal year of 2030. Therefore, there needs to be a future study and further extended observation of Japan’s energy structure. On the other hand, Japan is now expecting a greater environmental contribution from cleaner coal technology, such as coal gasification. However, concerns remain about whether Japan should go back to the coal era or not, especially under the global circumstance of coal divestment.

## Supplementary information

Appendix Fig 1 and 2
