# Supplementary material for: Japan prefectural emission accounts and socioeconomic data 2007 to 2015
Source: Sci Data. 2020 Jul 13;7:233. doi: 10.1038/s41597-020-0571-y (PMC7359347; doi:10.1038/s41597-020-0571-y)
Supplement: Supplementary file 1 — Appendix Fig 1 and 2 [file 41597_2020_571_MOESM1_ESM.pdf]

Appendix

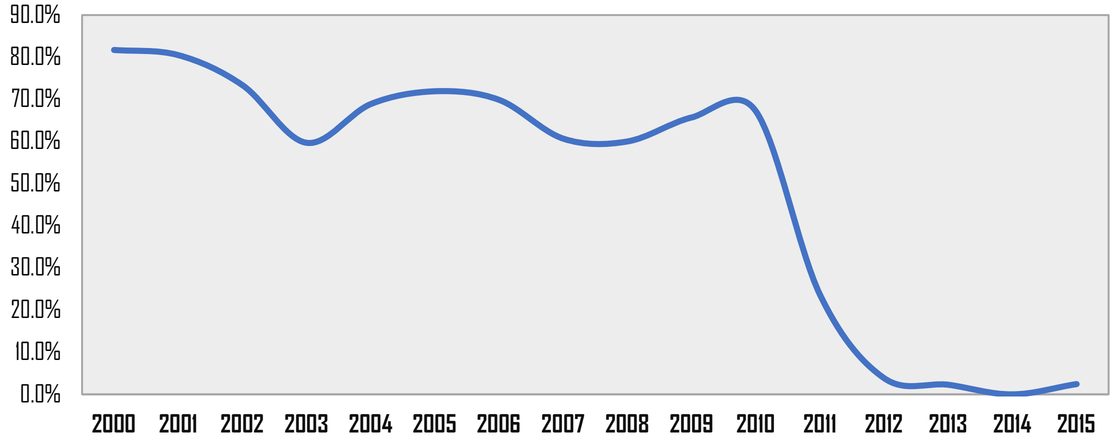

Fig.1 Capacity factor variation (Ratio) for nuclear power plant in Japan from 2000 to 2015

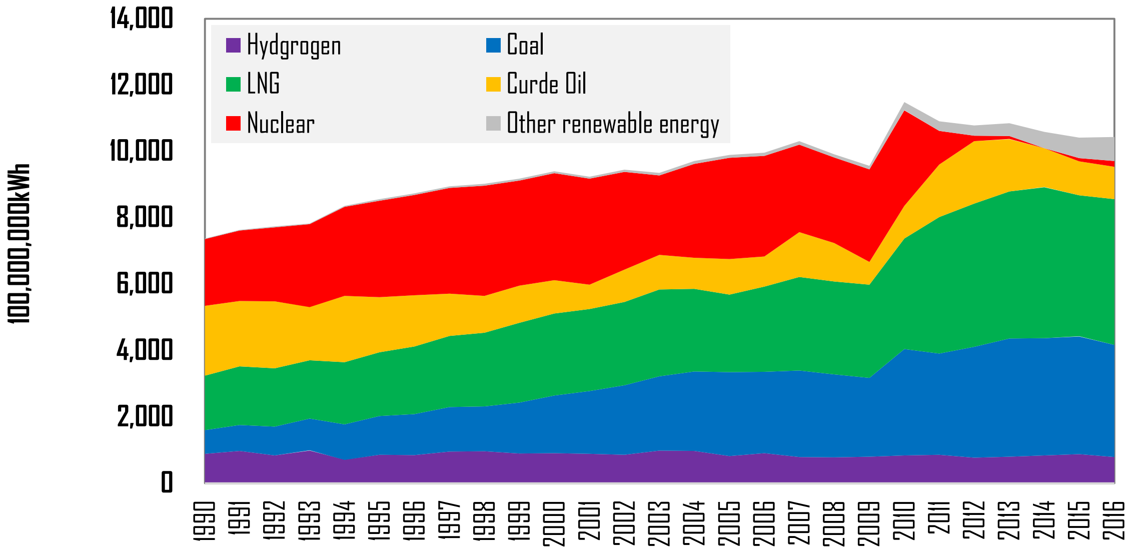

Fig.2 Energy mix of power generation in Japan from 1990 to 2015
